# The impact of anthelmintic drugs on weight gain of smallholder goats in subtropical regions

**DOI:** 10.1016/j.prevetmed.2018.08.014

**Published:** 2018-11-01

**Authors:** Paul R. Bessell, Neil D. Sargison, Kichuki Mirende, Ranjit Dash, Sanjay Prasad, Lamyaa Al-Riyami, Neil Gammon, Kristin Stuke, Roy Woolley, Miftahul Barbaruah, Philemon Wambura

**Affiliations:** a Epi Interventions, 17 Bangholm Avenue, Edinburgh, EH5 3AS, UK; b University of Edinburgh, Royal (Dick) School of Veterinary Studies, Easter Bush Veterinary Centre, Roslin, Midlothian, EH25 9RG, UK; c Department of Veterinary Microbiology and Parasitology, Faculty of Veterinary Medicine, Sokoine University of Agriculture, P.O. Box, 3019, Morogoro, Tanzania; d Gir Odisha Foundation, Kadei, Badachana, Jajapur, Odisha, India; e Vet Helpline India Pvt Ltd. H.No 31/32 Milanpur (Near Masjid No.1), Chandmari, Guwahati, 781021, Assam, India; f GALVmed, Doherty Building, Pentlands Science Park, Bush Loan, Penicuik, Edinburgh, EH26 0PZ, UK; g GALVmed, Galana Plaza, 4^th^ Floor, Wing C, Suite B, Galana Road, Kilimani, Nairobi, Kenya

**Keywords:** Anthelminthics, Goats, Parasitology, Weight, Body condition score, Smallholder

## Abstract

Helminth infections are recognised as a major impediment to the productivity of goats in smallholder production systems. We used a multilevel framework to estimate the impact that administration of locally available anthelminthic drugs can have on the weight gains of goats in smallholder settings in India and Tanzania.

We recruited 234 goats from 92 households from Odisha state in India and 253 goats from 15 households from Dodoma region in Tanzania. The goats were non-pregnant adult females, and from each household a minimum of two goats were recruited wherever possible. Each goat was randomly assigned to treatment with a locally available anthelminthic drug, or non-treatment. Each animal was tagged, weighed and had its body condition score (BCS) assessed. Animals were followed up after 28 and 56 days and re-weighed. To account for the local variations in exposure to helminths and for variations between households and herds, the data were analysed in a multilevel mixed model with herd in village as nested random effects.

Over the 56 days of study, the non-treated goats in India had gained a mean of 30.64 g per day (a daily gain of 0.23% baseline body weight) and in Tanzania 66.01 g per day (0.33% baseline body weight). From the mixed model, the treated goats in India gained a mean of 25.22 g per day more than non-treated goats, this is significantly greater than the weight gain in non-treated goats (p < 0.001). In Tanzania treated goats gained a mean of 9.878 g per day more than non-treated goats, which is also significantly greater than non-treated goats (p = 0.007). Furthermore, in India and Tanzania, goats with a lighter weight at the baseline survey gained greater amounts of weight. In both studies the BCS of the treated goats improved by a greater amount than the non-treated goats.

In this study we have demonstrated that in certain settings, the administration of anthelminthic drugs has a clear beneficial impact on goat weight.

## Highlights

• An assessment of the impacts of anthelminthic drugs on weights of goats in India and Tanzania
• Over 56 days, the treated goats in India gained weight at almost twice the rate of non-treated goats.
• Over 56 days the treated goats in Tanzania gained 15% more weight than non-treated goats
• In both areas, the body condition scores of treated goats also improved relative to non-treated goats.

## 1 Introduction

Smallholder farming is vital to agricultural production and the livelihoods of rural populations in subtropical countries, with goats being an important livestock species. Infections with helminths in goats are very common. Studies typically identify prevalences of infection that can be as high as 100% with very high burdens of infection in infected animals (Dixit et al., 2017; Rupa and Portugaliza, 2016; Sharma et al., 2016), but in other settings both the prevalence and burden of infection can be much lower (Haile et al., 2018). Helminth infections reduce weight gain, thus impacting on the time taken to reach target weights for slaughter or reproduction, and reducing the efficiency of conversion of nutritional inputs that are required for the animal to reach maturity (Sargison et al., 2017).

There are a number of anthelminthic drugs that are available off-the-shelf to smallholder farmers. Anthelminthics may be broad spectrum, or target specific helminth species, but the efficacy of some mode of action groups may be reduced by anthelminthic resistance (Kaplan and Vidyashankar, 2012). In smallholder settings, anthelminthics are typically administered *en masse* without determining the need or strategy for treatment. For many smallholders, the packaging size of products makes anthelminthic drugs inaccessible, hence programmes are being developed whereby anthelminthics are sold by members of the local community that are trained in administration of vaccines and anthelminthics alongside vaccines (Bessell et al., 2017). A key outcome of treatment with anthelminthics should be improved weight gain in treated animals, but in this context only a small number of studies have sought to estimate the impact that anthelminthic drugs have on the weight gain of small ruminants (Busin and Sargison, 2014; Coop et al., 1982; Sharma et al., 2016).

The objective of this study was to evaluate the impact of the administration of locally available anthelminthic drugs on the weights of smallholder animals where there is no prior diagnosis of infection. There are a number of factors that must be controlled within this study framework, such as differences in exposures, genetics and feeding regime. Many of these factors are clustered at the level of the herd and the village, hence a randomised controlled trial was used in which treatment with anthelminthics was randomised at the level of the individual animal, and within each study herd some animals were randomly assigned to treatment or to non-treatment.

## 2 Materials and methods

### 2.1 Study hypothesis

We assume that animals that are treated with anthelminthics will clear infections, acknowledging that there is a risk of reinfection, particularly with *Haemonchus* spp. Subsequently, in the period following treatment there will be a significantly greater rate of growth in the treated animals compared to the untreated animals. So we hypothesise that treating animals with anthelminthics has a statistically significant effect on weight gain over a 56 day period.

### 2.2 Study design

All animals were weighed at the time of treatment and then followed up and reweighed 28 and 56 days after the baseline. These time-points were selected to allow time for the drugs to have effect and the effect to be manifested in the body weight of the goats.

Any non-pregnant adult female goat was eligible for inclusion in the study, selected because adult females comprise the majority of the population, are at similar life stages, and will have more consistent histories of exposure to helminth infections. Pregnancy status was specified to avoid artificially altering the goat’s weight. The pregnancy status of the goats was checked by transabdominal palpation by animal health professionals at all surveys, but it remains possible that some early pregnancies may have been missed due to the low sensitivity of this technique (Karadaev, 2015). Goats were enrolled at the level of the herd. We defined a herd as a group of goats that were managed together and were under the same ownership.

All enrolled animals were given uniquely numbered ear tags to accurately identify each animal at the follow-up visits. To minimise the loss to follow-up of animals that are sold or are consumed a small financial incentive (approximately 3USD) was offered for each goat present at the end of the study that was under the ownership of the same household.

### 2.3 Study areas and timing

In order to compare a range of appropriate situations, we selected rural areas that have smallholder farmers whose animals comprise a substantial proportion of income and assets. Study sites were selected in Tanzania and India.

**Fig. 1:**
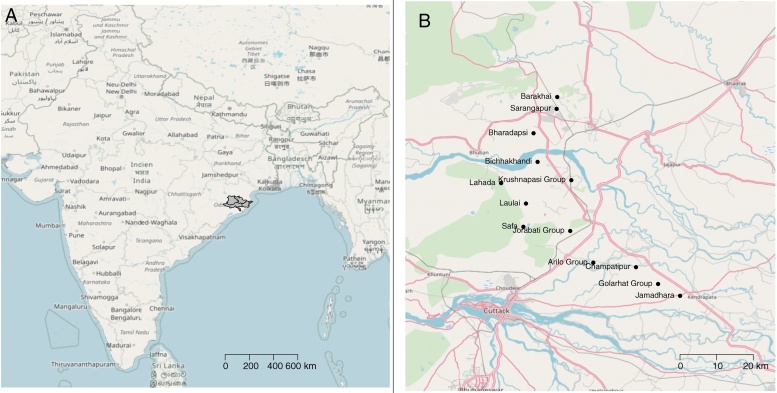
Map of India, showing the study districts of Cuttack, Dhenkanal, Jajpur and Kendrapara (A) and a zoomed map showing the study villages (B). The basemaps are from Open Street Maps (Open Street Map © OpenStreetMap contributors under a Creative Commons Attribution-ShareAlike 2.0 licence (CC-BY-SA)).

In India, the project was implemented in the districts of Cuttack, Dhenkanal, Jajpur, Kendrapara in the state of Odisha (Fig. 1). From 12 administrative blocks, a total of 18 villages were sampled. The baseline survey was conducted in December 2016, this is shortly after the wet season when the roundworm challenge is likely to be greatest. Importantly, it is also when the villages are accessible without any locally observed religious festivals that may have been a cause to slaughter animals.

**Fig. 2:**
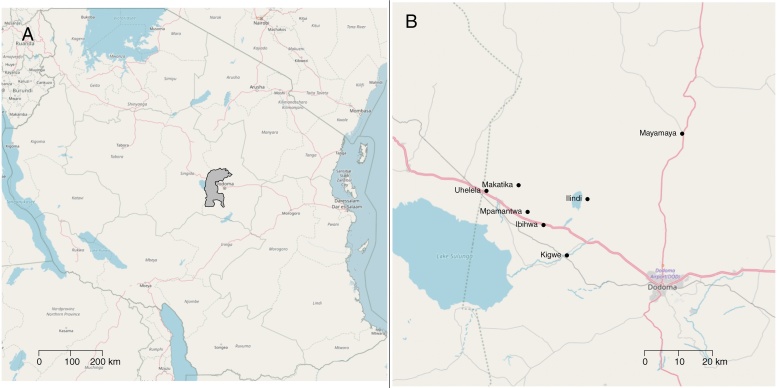
Map of Tanzania, showing the study districts of Bahi (A) and a zoomed map showing the study villages (B). The basemaps are from Open Street Maps (Open Street Map © OpenStreetMap contributors under a Creative Commons Attribution-ShareAlike 2.0 licence (CC-BY-SA)).

In Tanzania, the project was implemented in Bahi district of the normally semi-arid Dodoma region (Fig. 2). The baseline survey was conducted during the middle of January 2017, towards the end of a prolonged dry season (the rains normally start in December but were late). Due to difficulties accessing some villages in the Tanzania study, the endline survey was not necessarily exactly 56 days following baseline survey, but the exact numbers of days were recorded.

### 2.4 Household and animal enrolment

Households in the survey villages were visited to identify suitable candidates for enrolment into the study. Willing households were asked to sign an informed consent form (IC) that had been translated into the local language. The IC informed the farmer of the purpose and structure of the study and the terms of the financial incentive. If a household declined to sign the IC then another suitable household was enlisted. For each goat that was considered for enrolment:

1 The pregnancy status was checked by transabdominal palpation, animals that were identified as pregnant were not enrolled.
2 The animal was weighed using a platform apparatus.
3 The body condition score (BCS) was assessed using the standard three metric procedure – assessing the visual aspect, spinal process and the sternal fat on a 1:5 scale (Villaquiran et al., 2004) and an overall score from 1 to 5 assigned to the animal based on the consensus of the scores.
4 In India, a coin was tossed to assign the animal to treatment or non-treatment and in Tanzania, the Open Data Kit (ODK) Collect App (Hartung et al., 2010) was used to randomly assign animals to treatment or non-treatment, as this was the preference of the study teams.
5 If treatment was assigned then the animal was treated with a quantity of anthelminthic drug appropriate for the animal’s weight based on the manufacturer’s instructions, calculated by the ODK Collect App.
6 The animal was tagged and the data collected on smartphones using ODK Collect in both India and Tanzania.
7 At the 28 and 56 day surveys, the animals were reweighed, their pregnancy status checked, their BCS assessed and the data recorded on ODK Collect.
8 At the final survey, compensation was paid for all tagged animals that were present. At this point, anthelminthic was offered for all animals that were not treated at baseline.

### 2.5 Treatment and equipment

The anthelminthic drugs that were selected were locally available administered orally using a calibrated syringe to ensure that the correct dose rate was administered directly to the back of the mouth. Goats in India were treated with a single oral dose of closantel 15% oral solution (Zyclos^™^, Zydus AH), administered as per manufacturer’s instructions at 10 mg/kg (1 ml/15 kg). Farmers were informed of an arbitrary 42 day withdraw period for goat meat and instructed not to consume the goats’ milk, but these were meat goats and so this was not a major difficulty. Closantel is a narrow-spectrum anthelminthic and is effective against certain blood-feeding nematodes and trematodes, in particular *Haemonchus* spp. and *Fasciola* spp. There is no known resistance to closantel in Odisha State.

In Tanzania, goats were orally dosed with 7.5 mg/kg albendazole (Tramazole 10%; Univet Ireland Ltd, distributed by Ultravets Tanzania Ltd). Farmers were informed of the arbitrary 42 day withdrawal period for goat meat and instructed not to consume the goats’ milk at any time after treatment, but these were meat goats and so this was not a major difficulty. Albendazole is a broad spectrum benzimidazole anthelminthic, effective against most nematodes, but at these doses would have little efficacy against adult *Fasciola* and has little efficacy against cestodes. Furthermore, resistance to Benzimidazoles is widespread throughout the world (Kaplan and Vidyashankar, 2012).

### 2.6 Sample size

The required sample size was estimated as 234 (117 in each group). This was calculated using standard online sample size calculators as a superiority trial with a continuous outcome (daily weight change). We assumed that all adult animals had helminth infections and following treatment the mean daily growth rate in non-treated animals was 20 g/day and 26 g/day in treated animals (both with a standard deviation of 13 g/day) informed from a study in cattle which was used because we could not find sufficient parameters in the literature on goats (Keyyu et al., 2003, 2006, 2009a, 2009b). This gives a required sample size of 198 over which a 15% allowance was made for loss to follow-up, bringing the final sample size up to 234.

### 2.7 Statistical modelling

Data were analysed as a linear mixed model with the mean daily change in weight of the animal as the dependent variable and starting weight and treatment status of the animal the explanatory variables. To control for the effects of local village level exposures, any local parasite resistance and for herd level variations in genetics, husbandry and nutrition, a mixed model analysis was conducted in which the village and herd of the animal was included as random effects in a nested structure. Thus, the model for the daily change in weight (*y*) is:

$$y\approx a+b_{1}\left(start\;weight\right)+\;b_{2}\left(treatment\right)+\;\varepsilon$$

Where y is the mean daily change in weight of the goat between baseline and endline surveys modelled as:

$$y=\frac{weight\;at\;endline-weight\;at\;baseline}{days\;between\;baseline\;and\;endline}$$

*a* is the intercept and corresponds to the baseline change in weight among the study animals over the study and *b_n_* the estimate fitted for each explanatory variable. *b_1_* gives the change in weight relative to the baseline weight, so if animals have changed weight equally irrespective to their size then this will equal one. ε is the error term describing the variance due to the nested random effects of herd|village.

The model was fitted in the R statistical environment (R Core Team, 2017) using the lme4 package (Bates et al., 2015). P-values of the *a* and *b_n_* terms were calculated by taking corresponding values of the t-value from a normal distribution with mean zero and multiplying by two to give a two-tailed distribution. We also tested the effects of previous use of anthelminthics on goat weight and a non-linear quadratic effect of baseline weight by selecting the model with lowest value for Akaike information criteria (AIC).

Diagnostic plots of the residual versus the fitted values were checked for any structure. In addition, boxplots of the residuals at the village level were plotted to check for any unaccounted village level effects.

## 3 Results

### 3.1 Baseline data

In India, the sample size of 234 goats was enrolled from 14 villages that form 13 village groups (two of the villages are geographically indistinguishable from each other). The 234 goats were enrolled from 92 households (2.54 goats / herd; range 1–6). The majority were grazed during the day and given overnight shelter, but 9 herds were tethered, 50% of the herds gave their goats no supplementary nutrition. Knowledge of anthelminthics was minimal with only two respondents aware of anthelminthics and none having previously used anthelminthics.

In Tanzania, herd sizes were much larger and 253 goats were enrolled from 15 households (16.9 goats / herd, range 9–47). Of the 15 households, 10 (66.6%) had knowledge of anthelminthics and 8 respondents (53.3%) had previously used anthelminthics. All goats were grazed and given night shelter. Two herds (13.3%) were given some feed supplementation other than grazing and cut grass. In India and Tanzania, all goats enrolled were adult females and not in later stages of pregnancy.

### 3.2 Descriptive statistics

Extensive descriptive analysis of this study is presented in a non-reviewed report (Bessell, 2017). In India, a greater proportion of goats were randomly assigned to the treatment than the non-treatment group and there was a small difference in the weights and BCS at baseline (Table 1). Eight goats were lost to follow-up at 56 days because they had become pregnant during the study, but it is possible that a greater number of goats were in the early stages of pregnancy due to the poor sensitivity of the diagnostic method. The body weights of the goats in India were low, where the majority were of the small Black Bengal breed, whilst in Tanzania goats were local indigenous breeds (Table 1). The high standard deviation around the low body weights might also indicate that some immature females may have been recruited into the study.

**Table 1:** Summary of goats enrolled and treatment status.

	Total	Not treated	Treated
India			
Number of animals enrolled			
Baseline	234	101 (43.2%)	133 (56.8%)
28 days	234	101 (43.2%)	133 (56.8%)
56 days	226	97 (42.9%)	129 (57.1%)
Mean weight in kg (SD) at baseline	13.2 (4.8)	13.7 (4.9)	12.9 (4.7)
Mean BCS at baseline	2.08	2.15	2.02
Tanzania			
Number of animals enrolled			
Baseline	253	130 (51.4%)	123 (48.6%)
28 days	248	128 (51.6%)	120 (48.4%)
56 days	238	120 (50.4%)	118 (49.6%)
Mean weight in kg (SD) at baseline	20.1 (4.7)	19.4 (4.8)	20.9 (4.6)
Mean BCS at baseline	2.79	2.82	2.76

SD = standard deviation.

In Tanzania, a greater proportion of goats were assigned to non-treatment and 15 were lost to follow-up by not being present at the endline survey, or due to pregnancy (Table 1). Goats in Tanzania were around 7 kg heavier than in India (Table 1).

**Fig. 3:**
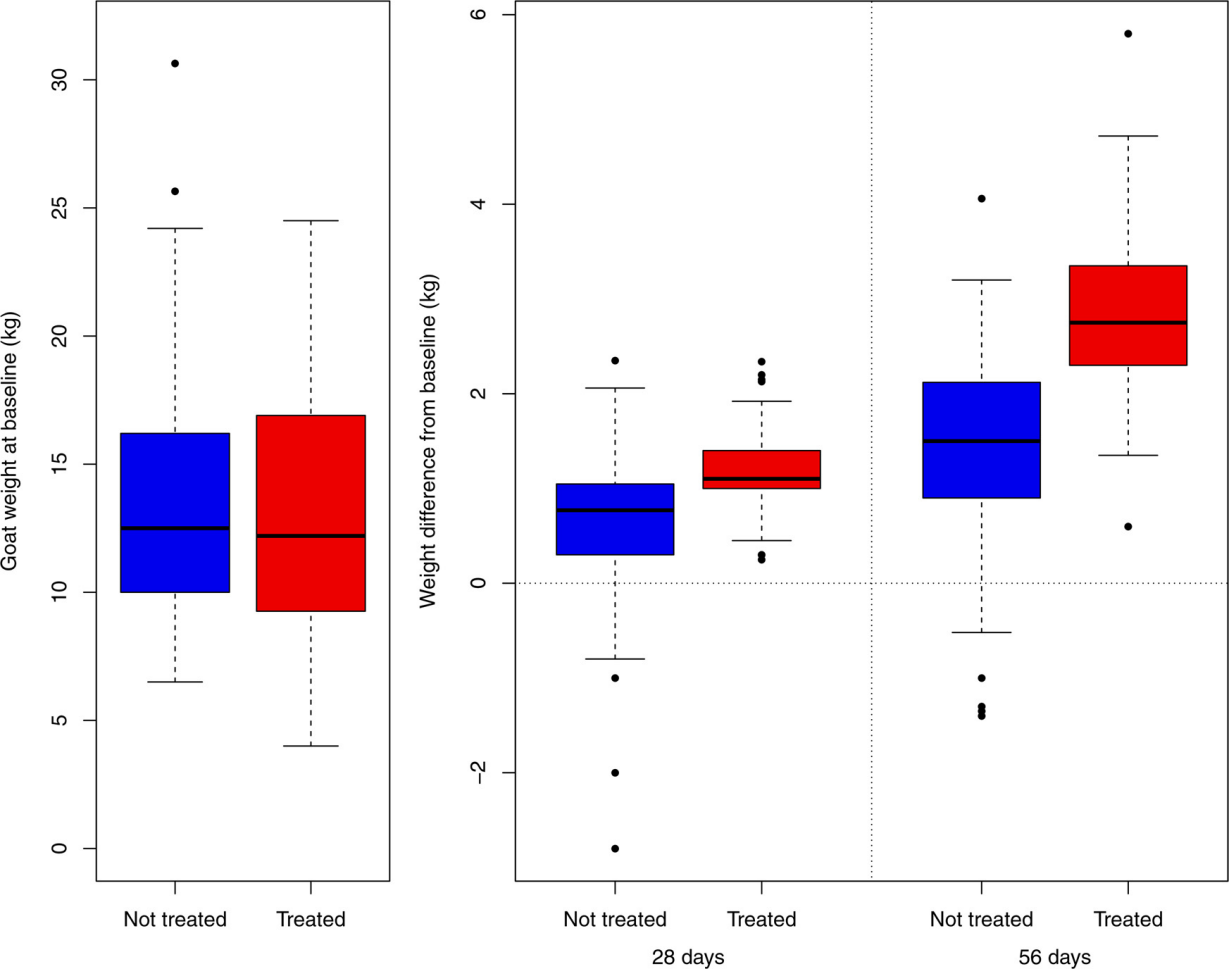
Boxplots of the goat weights in India at the baseline (left) and the difference in weights at the two follow-up surveys (right). The broken lines represent the smallest of 1.5 times the interquartile range or the range of the data. Outliers are indicated by a point. The 8 goats that were pregnant at the 56 day survey are removed from all analyses.

In India, both treated and non-treated goats gained weight over the course of the study (Fig. 3) but after 56 days, the treated goats had gained twice the amount of weight relative to the non-treated goats (2.85 kg compared to 1.41 kg) (Table 2). The corresponding change in BCS relative to the baseline after 56 days in non-treated goats was -0.03 and 0.40 in the treated goats (Table 2).

**Table 2:** The differences in weight and BCS of the goats in India at different the time points.

		28 day survey		56 day survey	
Difference compared to baseline		Not treated	Treated	Not treated	Treated
Overall weight change (kg)	Mean difference	0.611	1.186	1.410	2.846
	SD difference	0.790	0.377	1.037	0.808
	Difference range	−2.80–2.35	0.25–2.34	−1.40–4.06	0.60–5.80
Daily weight change	Mean daily change (g)	21.8	42.3	25.2	50.8
	Daily percentage change (%)	0.15	0.27	0.17	0.33
BCS (1:5 scale)	Mean difference	−0.027	0.269	−0.031	0.401
	SD difference	0.224	0.260	0.316	0.369
	Range	−1.0–0.33	−0.67–1.0	−1.33–0.67	−0.67–1.67

SD = standard deviation.

**Fig. 4:**
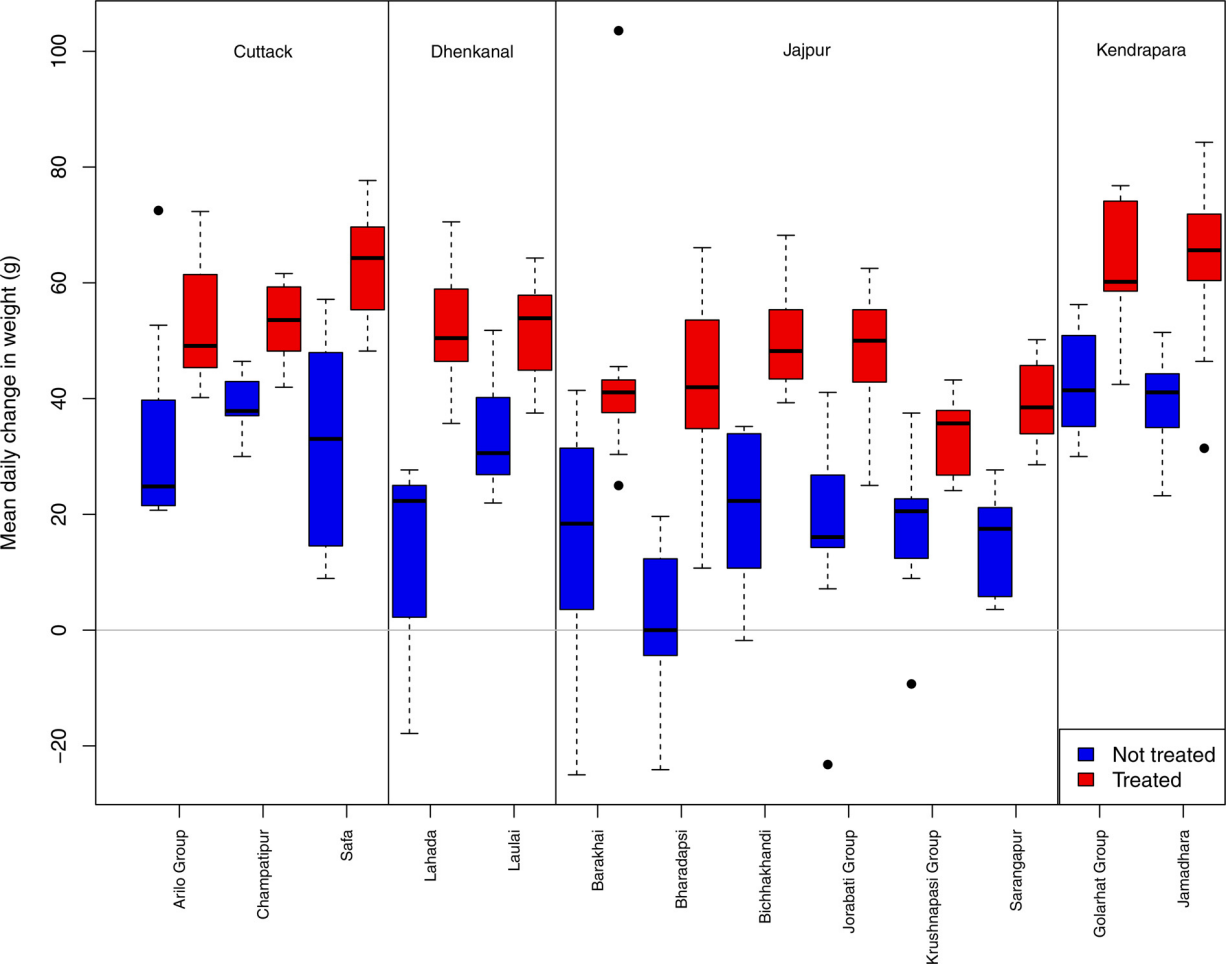
Change in goat weight over 56 days by village and district in India. The broken lines represent the smallest of 1.5 times the interquartile range or the range of the data. Outliers are indicated by a point.

Breaking down the mean daily change in weight in India by village and district shows no distinct pattern and there is a clear difference in the change in weight between the two groups in all villages (Fig. 4).

**Fig. 5:**
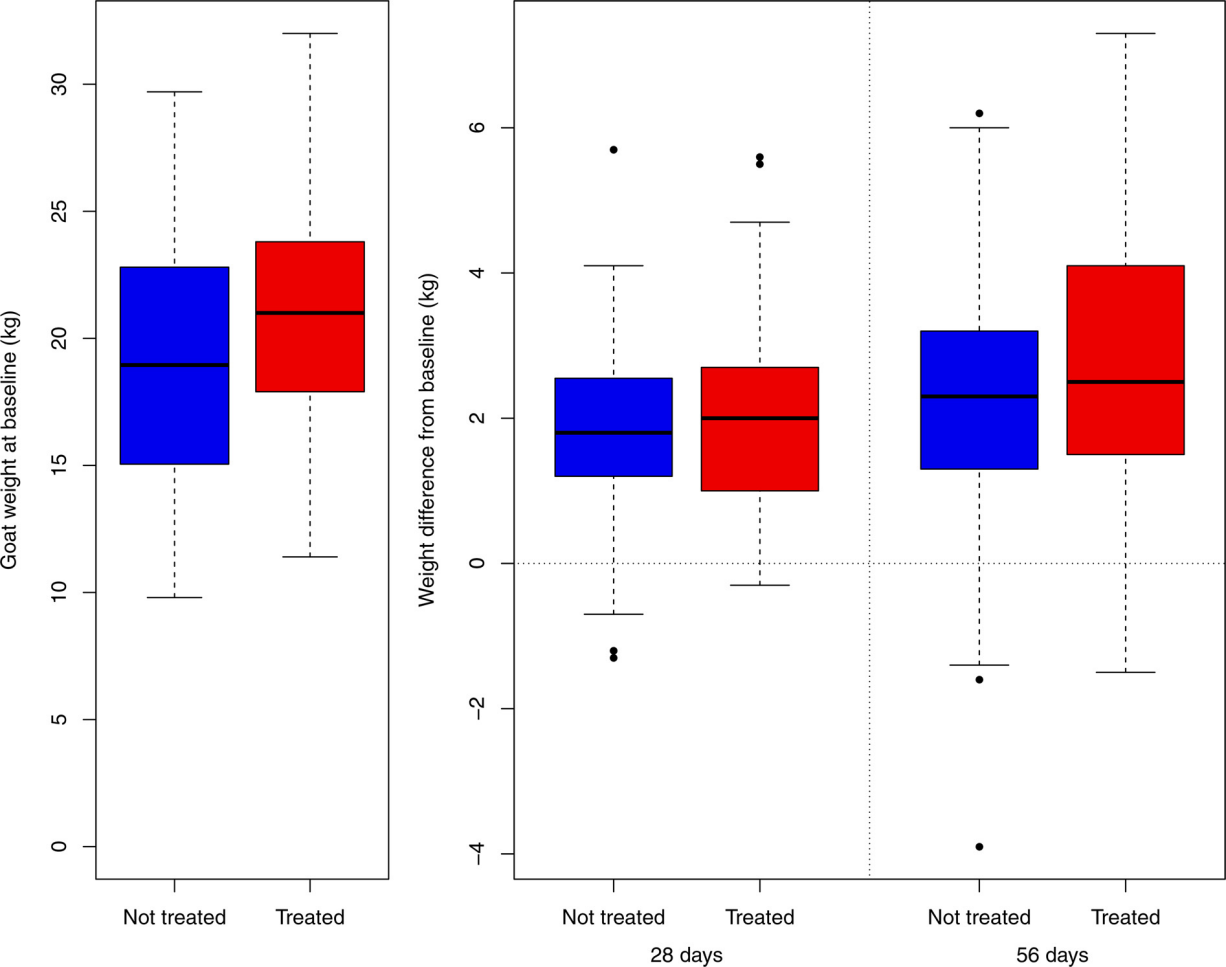
Boxplots of the goat weights in Tanzania at the baseline (left) and the difference in weights at the two follow-up surveys (right). The broken lines represent the smallest of 1.5 times the interquartile range or the range of the data. Outliers are indicated by a point. The 8 goats that were pregnant at the 56 day survey are removed from all analyses.

**Fig. 6:**
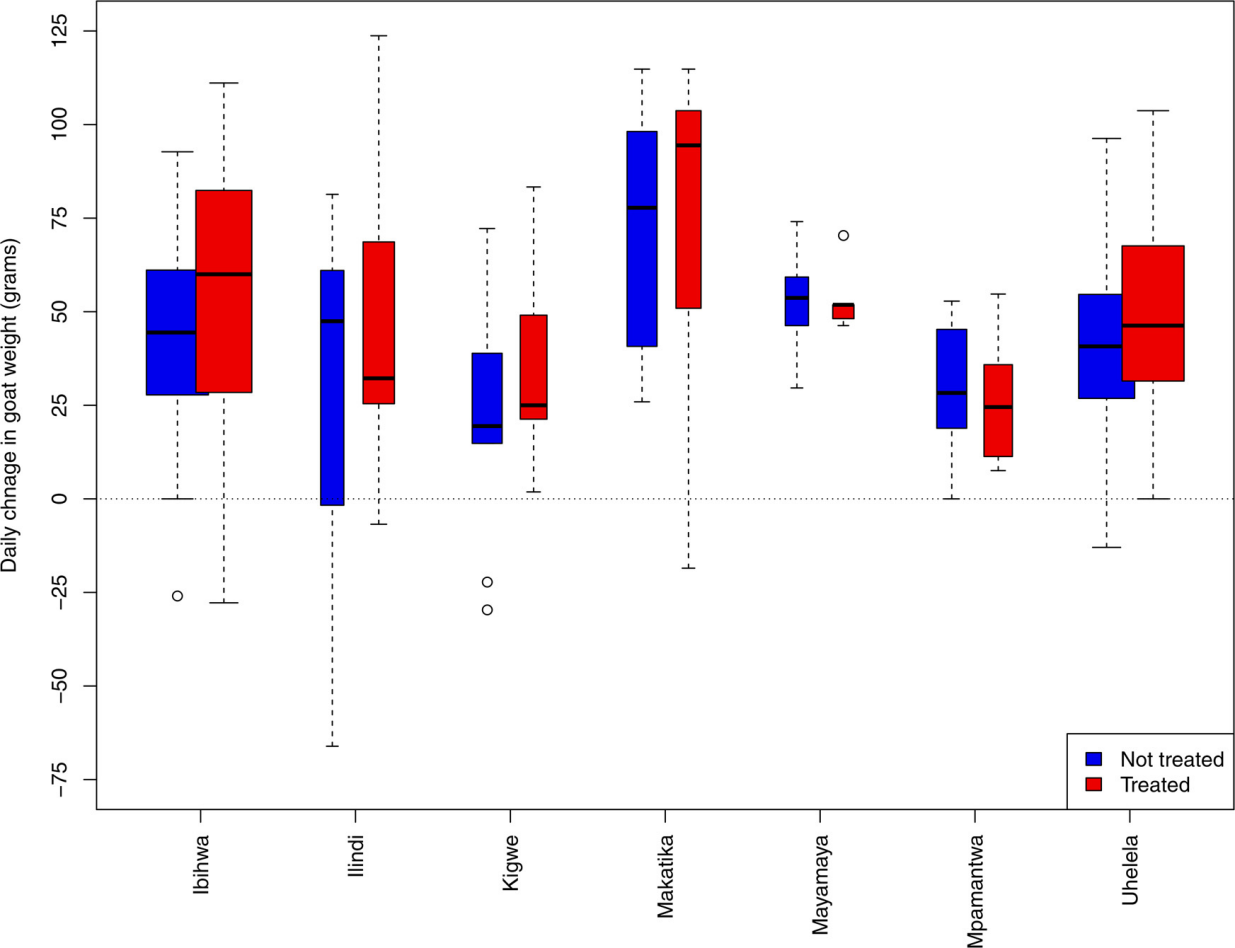
Boxplot showing the mean daily weight change by village in Tanzania. Note that due to the different numbers of goats enrolled in each village, the widths of the boxes represent the proportion of observations in that group. The broken lines represent the smallest of 1.5 times the interquartile range or the range of the data. Outliers are indicated by a point. The 8 goats that were pregnant at the 56 day survey are removed from all analyses.

In Tanzania, there was a visible difference between treated and non-treated goats in weights at both 28 and 56 days, but this is a smaller difference than in India (Fig. 5, Table 3). In total, 12 goats had lost weight over the course of the study. Weight gain in the non-treatment group in Tanzania was similar to the weight gains that were observed in India. BCS improved in both groups after 56 days, but the magnitude of the change was greater in the treated goats (Table 3). There is no clear pattern when analysed by village in Tanzania (Fig. 6).

**Table 3:** The differences in weight and BCS of the goats in Tanzania at the different time points.

		28 day survey		56 day survey	
Difference compared to baseline		Not treated	Treated	Not treated	Treated
Overall weight change (kg)	Mean difference	1.808	1.931	2.283	2.694
	SD difference	1.779	1.246	1.595	1.826
	Difference range	−9.6–10.6	−0.3–5.6	−3.9–6.2	−1.5–7.3
Daily weight change	Mean daily change (g)	64.0	68.5	42.1	49.5
	Daily percentage change (%)	0.33	0.33	0.22	0.24
BCS (1:5 scale)	Mean difference	0.258	0.288	0.342	0.517
	SD difference	0.510	0.681	0.642	0.949
	Range	−1.0–2	−1–2	−1–2	−1–3

SD = standard deviation.

**Fig. 7:**
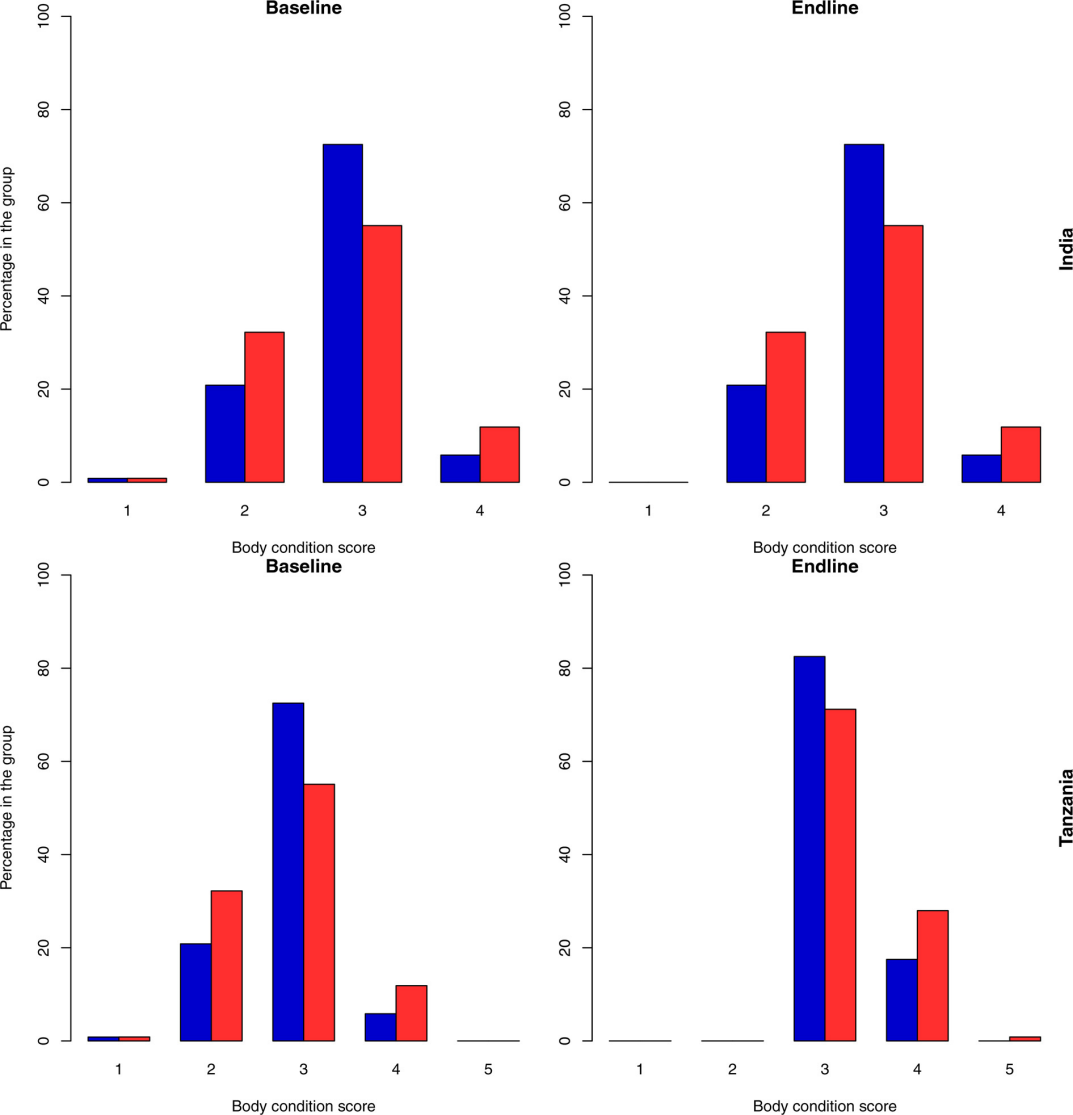
Barplots of the body condition scores at baseline and at endline. Blue bars represent the non-treated group and the red bars the treated groups (For interpretation of the references to colour in this figure legend, the reader is referred to the web version of this article).

In both study areas there was an overall improvement in the body condition scores between baseline and endline and this improvement was greater in Tanzania (Fig. 7). However, in India, there is a greater increase in the proportion of treated goats with a BCS of 3 compared to non-treatment goats (Fig. 7). In both sites, there were no goats with a BCS of 1 at endline and there were fewer with a BCS of 2.

### 3.3 Statistical modelling of the impact of treatment on weight change

In both studies, results from the mixed model show that the goats gained weight over the course of the study (Table 4), in India all goats gained a baseline 30.64 g/day compared to 66.01 g/day in Tanzania, both are significantly different to zero (p < 0.001) (Table 4). Heavier goats at baseline had significantly (p < 0.05) lower daily weight gain than lighter goats; for each kg of weight at baseline in India, the goat gained 0.4 g/day less, and 1.343 g/day less in Tanzania (Table 4). Treatment with anthelminthics also had a statistically significant effect, with treated goats in India gaining an additional 25.22 g/day (p < 0.001) and in Tanzania 9.878 g/day (p = 0.007) (Table 4). In India, the effect of tethering was analysed but this made no significant difference to the model fit. We tested the effect of including previous use of anthelminthics in the model for Tanzania as well as the inclusion of a quadratic term for the baseline weight, but these models had a larger AIC than the basic model and so were rejected. The residual plots of the models were checked and showed normality.

**Table 4:** Summary outputs of a multivariable linear mixed model of the average daily change in goat weight between the baseline and endline surveys.

	Estimate	Standard error	t-value	p-value
India				
Intercept	30.64 g	3.984g	7.691	<0.001
Baseline weight (kg)	−0.400 g/kg	0.202 g/kg	−1.978	0.048
Treatment Not treated Treated	– 25.22 g	– 1.849g	– 13.64	– <0.001
Tanzania				
Intercept	66.01g	9.340g	7.068	<0.001
Baseline weight (kg)	−1.343 g/kg	0.409 g/kg	−3.285	0.001
Treatment Not treated Treated	– 9.878 g	– 3.686 g	– 2.680	– 0.007

## 4 Discussion

Infection with gastro-intestinal parasites presents a persistent challenge to smallholder farming across the world. Anthelminthics are one of a number of strategies that can be used to minimise the impact and infection rates of helminths in goats. Other strategies that could be used in smallholder farm production systems include managed grazing, diversifying feed, manure management and breeding management (Gray et al., 2012). In this study we have used a multilevel framework for measuring the impact of treatment with anthelminthics on the weight gain and therefore productivity of goats.

In this study, none of the farmers in India had previously used anthelminthics, but a large proportion in Tanzania had previously done so, but previous use had no significant effect on weight gains seen in this study. In India, the administration of closantel had a major beneficial effect with the treated animals gaining almost twice the weight of the non-treated animals. Furthermore, in India, none of the treated animals lost weight. This indicates that there was a high prevalence of *Fasciola* spp or *Haemonchus* spp and that closantel was an effective treatment that cleared at least some of these infections. It is more likely that the observed change in weight was due to clearing an infection with *Fasciola* spp as the rate of reinfection with *Fasciola* spp can be much slower than with *Haemonchus* spp.; this depends on the seasonality of metacercarial or infective larval challenge. The study was conducted immediately after the wet season at a time when there may be a large *Haemonchus* larval challenge that has greatest impact during the period immediately following infection. The consistent response to closantel treatment in all of the study villages indicates that the infections were commonplace across the area and that the drug was effective against at least some parasites (Fig. 4) but we do not know with certainty the contribution of any species to the helminth burdens. Given additional time and resources we could have done further work to evaluate the locally circulating helminth species through collecting and analysing a series of faecal samples. However, the resources available for this study prevented this and as such the study is carried out as seen by the farmer – effectively blind to the pathogens involved.

In Tanzania the effect of treating with a broad-spectrum anthelminthic on liveweight gain was more modest, with all animals gaining a mean of 66.01 g per day before the baseline bodyweight effect is taken into consideration and treated animals gaining an additional 9.878 g per day (Table 4). The lower impact of anthelminthics in Tanzania compared to India could be due to a number of factors:

1 The prevalence of infections with helminth species is much lower than in India.
2 This study was towards the end of a prolonged dry season when the goats may have been under some considerable stress and their *Haemonchus* parasite populations may have entered a state of hypobiosis, having a lower impact on production.
3 At the dosage used in this study albendazole will be effective against nematodes but may not be effective against trematodes. During a long dry season such as this, there would be a low level of nematode infection, so clearing these would have small effect. Given the dosage used, then even if this area had a high prevalence of *Fasciola* infection then these infections may not have been cleared.
4 The anthelminthic used in Tanzania was ineffective against the parasite populations that were circulating locally. (Gray et al., 2012, 2008; Wong and Sargison, 2017), or the quality of the product used in Tanzania was compromised.
5 The impact of anthelminthics has been underestimated due to the sample size or small number of households that were enrolled. Only 15 households were enrolled in Tanzania, compared to 92 households in India. It remains possible that a larger number of sampled households would have yielded a greater effect. The numbers of households that were recruited in Tanzania reflected the size of herds and the logistics of moving between the sparsely distributed households in the study area in Tanzania.

In both study areas the daily change in bodyweight was greater amongst animals that had lower weight at baseline, underlining the value of treating helminth infections in animals of a lighter weight. Furthermore, the impact of chronic haemonchosis is known to be greater among lighter animals (Besier et al., 2016), giving greater benefit of deworming. The positive change in BCS observed here among the treated animals suggests that the treated animals were putting on body fat and supports the differential changes in live weight gain that were seen. Body condition score is critical both for assessing the general health of the animal and when the value of the goat is assessed at sale. In this study, a greater proportion of goats had a BCS of 3 at the end of the study, with 3 being recognised as the optimal score in terms of animal health with no animals judged to have a score of 1 which corresponds to a severely emaciated and unhealthy state (Fig. 7).

The observed weight gains in this study among almost all animals was an unexpected result from the study. Whilst some immature animals were certainly recruited, particularly in India, and this may explain some of the observed weight gains but does not explain most of the change as even the heavier goats at baseline gained weight. This result may be down to seasonal variations in management between the areas, or if the farmers adopted the practice of fattening the goats for slaughter.

These results show that prophylactic treatment with anthelminthics can have a positive effect on a goat’s productivity, leading to a larger carcass weight that will require lower inputs. The most likely deployment of these anthelminthics among farmers would be that they are sold through community workers (sometimes known as vaccinators) to overcome challenges of packaging size. However, consideration must be given to the timings of the intervention and the drugs that are used in order to maximise their impacts. This requires further work to understand the optimal timings of treatments for each area and an economic analysis should consider the cost of the treatment and savings in terms of the cost of inputs.

The improved BCS and weights of the goats will lead to a number of beneficial impacts to the farmer. Sale value is normally assessed through weight and evaluation of the body condition, or if the goat is slaughtered for home consumption then greater nutritional value will be achieved. More widely, the animal will require lower inputs in order to reach maturity and thus potentially make goat rearing more economical. Further analyses could consider the impact of anthelminthics on pregnancy rates and kid growth given improved milk production. However, many of these differences would need to be evaluated in a more holistic study that includes metrics for evaluating the economics of goat production.

## Sources of funding

This is based on research funded in part by the Bill & Melinda Gates Foundation and with UK Aid from the UK Government through GALVmed. The findings and conclusions contained within are those of the authors and do not necessarily reflect positions or policies of the Bill & Melinda Gates Foundation or the UK Government.
